# Prognostic Impact of Concomitant Genomic Alterations in FGFR2‐Positive Cholangiocarcinoma Treated With Pemigatinib

**DOI:** 10.1111/liv.70813

**Published:** 2026-07-24

**Authors:** Carolina Liguori, Riccardo Giampieri, Blandine Delaunay, Giada Pinterpe, Adelaide Mazzocca, Antoine Hollebecque, Jean Frederic Blanc, Mohamed Bouattour, Eric Assenat, Meher Ben Abdelghani, Matthieu Sarabi, Monica Niger, Caterina Vivaldi, Mario Mandalà, Andrea Palloni, Maria Bensi, Silvio Ken Garattini, David Tougeron, Pierre Combe, Massimiliano Salati, Margherita Rimini, Andrea Casadei‐Gardini, Chiara Alessandra Cella, Marco Tucci, Anna Diana, Elena Mori, Raffaella Longarini, Pascal Artru, Gael Roth, Ludovic Evesque, Agathe Vienne, Anthony Turpin, Sandrine Hiret, Vincent Bourgeois, Camille Herve, Rodolphe Paulon, Marion Stacoffe, David Malka, Matthieu Delaye, Julien Edeline, Astrid Lievre, Rosine Guimbaud, Rita Balsano, Nadim Fares, Rossana Berardi, Alessandro Parisi

**Affiliations:** ^1^ Clinica Oncologica e Centro Regionale di Genetica Oncologica Università Politecnica delle Marche, Azienda Ospedaliero‐Universitaria delle Marche Ancona Italy; ^2^ Digestive Oncology Department Centre Hospitalier Universitaire de Toulouse – Hopital Rangueil Toulouse France; ^3^ Département d'Innovation Thérapeutique et Essais précoces (DITEP) Gustave Roussy Villejuif Cedex France; ^4^ Oncology Digestive Unit, Hôpital haut‐Lévêque CHU de Bordeaux Bordeaux France; ^5^ Liver Oncology and Therapeutic Innovation Functional Unit Beaujon Hospital APHP Clichy France; ^6^ Medical Oncology, ICM – Institut du Cancer de Montpellier Montpellier Cedex France; ^7^ Oncology Department ICANS – Institut de Cancérologie Strasbourg Europe Strasbourg France; ^8^ GI Oncology Department Medical Oncology, Centre Léon Bérard Lyon France; ^9^ GI Oncology Department Hôpital privé Jean Mermoz Lyon France; ^10^ Medical Oncology Department Fondazione IRCCS Istituto Nazionale dei Tumori di Milano Milan Italy; ^11^ Department of Translational Research and New Technologies in Medicine and Surgery University of Pisa Pisa Italy; ^12^ Unit of Medical Oncology University of Perugia Perugia Italy; ^13^ Medical Oncology IRCCS Azienda Ospedaliero‐Universitaria di Bologna Bologna Italy; ^14^ Oncologia Medica, Comprehensive Cancer Center Fondazione Policlinico Universitario Agostino Gemelli–IRCCS Roma Italy; ^15^ Università Cattolica del Sacro Cuore Roma Italy; ^16^ Department of Oncology Academic Hospital of Udine ASUFC Udine UD Italy; ^17^ Department of Gastroenterology and Hepatology Poitiers University Hospital Poitiers France; ^18^ Medical Oncology, CORT37 Pôle Santé Léonard de Vinci Chambray‐lès‐Tours France; ^19^ Division of Oncology, Department of Oncology and Hematology University Hospital Modena, Modena Cancer Centre Modena Italy; ^20^ Clinical and Experimental Medicine University of Modena and Reggio Emilia Modena Italy; ^21^ Department of Oncology, Vita‐Salute University San Raffaele IRCCS San Raffaele Hospital Milan Italy; ^22^ Division of Gastrointestinal Medical Oncology and Neuroendocrine Tumors European Institute of Oncology, IEO IRCCS Milan Italy; ^23^ Department of Interdisciplinary Medicine, Oncology Unit University of Bari “Aldo Moro” Bari Italy; ^24^ UOC Oncologia – Ospedale del Mare Naples Italy; ^25^ Department of Medical Oncology New Hospital of Prato S. Stefano Prato Italy; ^26^ Fondazione IRCCS San Gerardo dei Tintori Monza Italy; ^27^ Univ. Grenoble Alpes/Hepato‐Gastroenterology and Digestive Oncology Department CHU Grenoble Alpes/Institute for Advanced Biosciences, CNRS UMR 5309‐INSERM U1209 Grenoble France; ^28^ Medical Oncology Department Centre Antoine‐Lacassagne Nice France; ^29^ Oncology Department CHU Sud Réunion Saint Pierre France; ^30^ Medical Oncology Department Hospital Claude Huriez Lille France; ^31^ Oncology Department ICO Institut de Cancerologie de l'Ouest René Gauducheau Saint‐Herblain France; ^32^ Digestive Oncology Hopital duchenne Boulogne‐sur‐Mer France; ^33^ Digestive Oncology Groupe Hospitalier Mutualiste Grenoble France; ^34^ Medical Oncology Clinique du Sidobre Castres France; ^35^ Medical Oncology CHRU Hopitaux de Tours – Hopital Bretonneau Tours Cedex France; ^36^ Medical Oncology Institut Mutualiste Montsouris Paris France; ^37^ GI Oncology, Medical Oncology Department Curie Institute Paris France; ^38^ Medical Oncology Department Centre Eugene‐Marquis Rennes France; ^39^ Department of Gastroenterology CHU de Rennes – Hopital Pontchaillou Rennes Cedex France; ^40^ Department of Biomedical Sciences Humanitas University Pieve Emanuele Milan Italy; ^41^ Medical Oncology and Hematology Unit Humanitas Cancer Center, IRCCS Humanitas Research Hospital Rozzano Milan Italy

**Keywords:** BTC, cholangiocarcinoma, *FGFR2* fusions/rearrangements, genomic alterations, prognostic factors, real‐world data, targeted therapy

## Abstract

**Background & Aims:**

Anti‐FGFR therapies changed the treatment landscape for patients with previously treated, advanced, or metastatic cholangiocarcinoma (CCA) harbouring *FGFR2* fusions/rearrangements. However, primary resistance to FGFR inhibitors remains a key challenge. In the present real‐world study, we aimed to evaluate the prognostic impact of concomitant GAs in patients with *FGFR2*‐positive CCA treated with pemigatinib.

**Methods:**

This study included PEMIREAL‐PEMIBIL patients treated with pemigatinib in second or later lines. Only patients who underwent extensive DNA‐ and/or RNA‐based next‐generation sequencing (NGS) analysis were considered. Primary endpoint was progression‐free survival (PFS), with overall response rate, disease control rate and overall survival (OS) as secondary endpoint. OS and PFS were calculated by Kaplan–Meier and log‐rank test. Multivariate analysis used cox‐regression model. Level of statistical significance *p* was 0.05.

**Results:**

Of 72 patients of PEMIREAL‐PEMIBIL, 63 patients had evaluable NGS data, with concomitant GAs identified in 28 patients (44.4%). The most frequently observed concomitant GAs involved *BAP1* (7/63, 11.1%), *CDKN2A* (7/63, 11.1%), *TP53* (6/63, 9.5%), *CDKN2B* (5/63, 7.9%), *PTEN* (3/63, 4.7%) and *IDH1* (1/63, 1.5%). A significantly shorter PFS was observed in patients with *CDKN2A* mutations compared to *CDKN2A* wild‐type tumours (4.79 vs. 8.66 months, *p* = 0.0011, HR: 3.48 95% CI: 0.91–13.24), and similarly in patients with *BAP1* mutations compared to *BAP1* wild‐type tumours (5.97 vs. 8.52 months, *p* = 0.025, HR:2.55 95% CI: 0.72–9.00). No significant differences in OS were observed.

**Conclusions:**

Our results support the negative prognostic role of *BAP1* and *CDKN2A* GAs on PFS in patients with locally advanced or metastatic CCA with *FGFR2* gene fusion/rearrangement treated with pemigatinib in a real‐world setting.

## Lay Summary

Pemigatinib is a targeted therapy used for advanced or metastatic cholangiocarcinoma with FGFR2 alterations. In this study, we observed that patients whose tumours also carried specific additional genetic changes—particularly in the *BAP1* and *CDKN2A* genes—experienced faster disease progression while on treatment. These results suggest that such co‐existing genetic alterations may help identify which patients are less likely to benefit from pemigatinib.

**Abbreviations**
CCA: cholangiocarcinoma
CR: complete response
dCCA: distal cholangiocarcinoma
DCR: disease control rate
DOR: duration of response
EAP: Expanded Access Program
eCCA: extrahepatic cholangiocarcinoma
FOLFOX: 5‐fluorouracil and oxaliplatin
GAs: genomic alterations
iCCA: intrahepatic cholangiocarcinoma
MSI: microsatellite instability
NGS: next‐generation sequencing
ORR: overall response rate
OS: overall survival
pCCA: perihilar cholangiocarcinoma
PFS: progression‐free survival
PR: partial response
SD: stable disease
SNVs: single‐nucleotide variants
TMB: tumour mutational burden

## 1 Introduction

Cholangiocarcinoma (CCA) is a rare but aggressive malignancy of the bile ducts, accounting for approximately 3% of all gastrointestinal tumours [1]. Based on anatomical location, it is classified into intrahepatic (iCCA), perihilar (pCCA) and distal (dCCA) subtypes. Over recent decades, the incidence and mortality of CCA have been rising globally, with a notable increase in iCCA [2].

Despite advancements in treatment over the past years, the prognosis for CCA remains poor. Most patients present at an advanced stage, and only 20%–30% are eligible for curative surgery [3]. For unresectable or metastatic CCA, the standard first‐line systemic treatment consists of chemotherapy with gemcitabine and cisplatin in combination with immunotherapy using PD‐L1/PD‐1 inhibitors, such as durvalumab [4] and pembrolizumab [5]. For second‐line treatment, 5‐fluorouracil and oxaliplatin (FOLFOX) remain the standard for all CCA patients, but its efficacy is limited [6].

Recent genomic studies have highlighted the molecular heterogeneity of CCA along the biliary tree [7]. Next‐generation sequencing (NGS) has provided insights into the complex genomic landscape of CCA, identifying the most frequently altered genes in iCCA (*ARID1A*, *BAP1*, *BRAF*, *ERBB2*, *FGFR2*, *IDH1/2*, *KRAS*, *NRAS*, *PRBM1*, *PIK3CA*, *SMAD4*, *TP53*) and extrahepatic cholangiocarcinoma (eCCA) (*ARID1A*, *ERBB2*, *KRAS*, *SMAD4*, *TP53*) [1, 8, 9]. Notably, up to 40% of patients with CCA, particularly those with iCCA, harbour genomic alterations that are potentially targetable with molecularly matched therapies [10]. Compared to standard, non‐matched treatments, molecularly guided therapies have been associated with higher response rates and improved survival outcomes [11, 12], particularly in iCCA, where *IDH1* mutations occur in 10%–15% of cases and *FGFR2* alterations in approximately 10% of cases [7].

*FGFR2* is part of the *FGFR* gene family, which includes four receptor tyrosine kinases (FGFR1‐4). Its activation depends on ligand‐induced dimerization, which initiates downstream signalling pathways like RAS–ERK. However, oncogenic *FGFR2* activation occurs independently of ligands due to catalytic activation driven by gene amplification, short variants, insertions/deletions, or fusion protein formation. Among these mechanisms, chromosomal rearrangements are the most frequent (9.4%), with *FGFR2* fusion events often involving partners such as *BICC1*, *SORBS1*, *AHCYL1* and *CCDC6* [7].

Pemigatinib was the first targeted therapy approved by the FDA and EMA for patients with CCA harbouring *FGFR2* fusions or rearrangements. The phase II, multicentre, open‐label FIGHT‐202 trial (NCT02924376) assessed safety and antitumour efficacy of pemigatinib in previously treated patients with locally advanced or metastatic cholangiocarcinoma. In patients with *FGFR2* fusions or rearrangements, by final analysis [13], the overall response rate (ORR) was 37.0% (95% CI: 27.9–46.9). With a median follow‐up of 45.4 months, responses proved durable, median duration of response (DOR) was 9.1 (95% CI: 6.0–14.5) months. Median progression‐free survival (PFS) and median overall survival (OS) were 7.0 (95% CI: 6.1–10.5) months and 17.5 (95% CI: 14.4–22.9) months, respectively.

Currently, no phase III/IV data are available. The phase III FIGHT‐302 trial (NCT03656536), which compared first‐line pemigatinib with CisGem, was prematurely discontinued due to low recruitment.

Silverman et al. [14] conducted a post hoc analysis using genomic profiling and clinical data from patients prescreened and enrolled in the FIGHT‐202 trial. The study investigated the genomic factors influencing response to pemigatinib, particularly the role of co‐occurring genetic alterations (GAs). The analysis found no clear correlation between specific *FGFR2* fusion partners and treatment outcomes. Patients with *FGFR2* rearrangements had fewer GAs (3.7 alterations per patient) compared to non‐rearranged patients (4.7 alterations per patient), with frequent co‐alterations in tumour‐suppressor genes such as *BAP1* (38.4%), *CDKN2A* (21.7%) and *PBRM1* (9.4%). While *BAP1* mutations did not significantly affect the ORR, they were associated with a trend towards shorter median PFS (6.9 vs. 9.1 months, *p* = 0.06). Patients with mutations in *CDKN2A/B* or *PBRM1* had lower ORRs (23.8% compared to 38.4% for *CDKN2A/B*) and significantly shorter PFS—6.4 months versus 9.0 months for *CDKN2A/B* mutations (*p* = 0.03) and 4.7 months versus 7.0 months for *PBRM1* mutations (*p* = 0.05). Notably, the nine patients with *TP53* mutation had no objective responses and significantly reduced PFS (2.8 vs. 9.0 months, *p* = 0.0003). Loss of tumour‐suppressor genes (*BAP1*, *CDKN2A/B*, *TP53*, *PBRM1*, *ARID1A*, *PTEN*) was associated with significantly reduced PFS (6.8 vs. 11.7 months, *p* = 0.0003) but did not impact ORR. Few patients had co‐mutations in oncogenic driver genes such as PIK3CA (*n* = 9) and IDH1 (*n* = 5), with no significant effect on outcomes. In the same patient cohort analysed in FIGHT‐202 [13], TP53 and PBRM1 mutations were also associated with shorter OS: 9.8 vs. 19.0 months for TP53 (*p* = 0.002), and 12.0 vs. 19.0 months for PBRM1 (*p* = 0.007). In contrast, CDKN2A, CDKN2B and BAP1 mutations did not significantly impact OS. These data suggest that CDKN2A and CDKN2B mutations may have a predominantly predictive, rather than prognostic, negative role for these GAs. These findings preliminarily suggested that co‐occurring GAs, particularly in tumour‐suppressor genes, may contribute to primary resistance to pemigatinib, highlighting the importance of comprehensive genomic profiling in *FGFR2*‐rearranged cholangiocarcinoma.

We previously supported the efficacy, activity, and safety of pemigatinib in a large real‐world population from two multicentre observational retrospective cohort studies independently conducted in France (PEMIBIL) and Italy (PEMIREAL) and including 72 patients with CCA who received pemigatinib as a second‐ or later‐line systemic treatment [14]. As of the data cut‐off, ORR was 45.8%, with a median DOR of 7.0 months (95% CI: 5.8–9.3). Over a median follow‐up of 19.5 months, median PFS was 8.7 (95% CI: 7.3–11.8) months, while median OS reached 17.1 (95% CI: 12.7‐NA) months.

Capitalizing from real‐world data of the PEMIBIL and PEMIREAL cohort studies, with the aim to further explore factors of primary resistance in patients treated with FGFR2 inhibitors, we investigated the prognostic and predictive role of co‐occurring GAs in *FGFR2*‐positive patients treated with pemigatinib.

## 2 Materials and Methods

### 2.1 Study Population

Details on the study design, eligibility criteria, and efficacy and safety findings of PEMIREAL‐PEMIBIL have been previously published [15].

PEMIREAL and PEMIBIL were two multicentre, observational, retrospective cohort studies independently conducted in Italy and France, respectively. Both included patients diagnosed with previously treated, unresectable, locally advanced or metastatic CCA harbouring *FGFR2* fusions or rearrangements and treated with pemigatinib as second or further line of systemic treatment either within or outside the European Expanded Access Program (EAP) sponsored by Incyte (Wilmington, DE, USA), in accordance with each country's drug reimbursement policies and timelines. Incyte was not involved in the study's design, data collection, or analysis.

Medical records from patients treated at 14 Italian and 25 French oncology centres between July 2020 and September 2022 were reviewed. The key inclusion criteria for both cohorts were: (a) adult patients with confirmed locally advanced or metastatic CCA harbouring *FGFR2* fusion or rearrangement; (b) availability of clinical and pathological data, including treatment patterns and outcomes; (c) prior administration of at least one systemic therapy for advanced/metastatic disease and at least one cycle of pemigatinib.

For the present analysis, an additional inclusion criterion was considered: (d) availability of extensive DNA or RNA‐based next‐generation sequencing (NGS) analysis for molecular characterization.

This study received approval from the respective local Ethics Committees of all participating institutions. It adheres to Good Clinical Practice guidelines, the Declaration of Helsinki, and applicable local regulations, including Regulation (EU) 2016/679 of the European Parliament and the Council of April 27, 2016, concerning the protection of personal data. Specifically, this analysis represents a joint evaluation of two independently approved retrospective observational studies: the Italian PEMIREAL study (approved by ‘Comitato Etico Regione Marche’ in December 2022, protocol number 325–2022) and the French PEMIBIL study (protocol number RnIPH 2022‐105).

### 2.2 Study Endpoints

This study analysed the genomic profiling and clinical outcomes of patients enrolled in the PEMIREAL‐PEMIBIL study to explore the relationship between co‐occurring GAs and clinical activity and efficacy of pemigatinib in patients with CCA harbouring *FGFR2* fusions or rearrangements. PFS was the primary endpoint, while ORR, disease control rate (DCR), PFS and OS were secondary endpoints. The predictive and prognostic impact of identified variants was assessed by comparing outcomes between mutant and wild‐type patient groups.

Treatment responses were evaluated in accordance with RECIST 1.1 criteria. ORR was defined as the proportion of patients experiencing an objective response (complete response—CR or partial response—PR) as the best response, as reported by the local investigator. DCR was defined as the proportion of patients experiencing CR, PR or stable disease (SD) according to RECIST 1.1, as per local investigator assessment. PFS was defined as the time from the beginning of treatment to the first evidence of objective disease progression or death of the patient for any cause, whichever occurred first. Determination of disease progression was based on the measurements reported by the investigator. OS was defined as the time from the start of treatment to the date of death for any cause. For patients still alive at the time of analysis, OS was censored at the last date of follow‐up.

### 2.3 Statistical Analysis

OS and PFS were calculated by the Kaplan–Meier method.

Statistical differences in PFS and OS distributions in the presence or absence of GAs were calculated using the log‐rank test. Exact Fisher test was employed to compare ORR and DCR between groups. The alpha level for all analyses was set to a *p* value < 0.05. Statistical analyses were performed using R software (version 4.3.0).

## 3 Results

### 3.1 Patients and Disease Characteristics

From July 2020 to September 2022, 63 patients among the 72 patients enrolled at 14 Italian and 25 French Oncology Units, were included (CONSORT diagram of patients' selection and inclusion in Figure S1). The main patients' and disease characteristics are summarized in Table 1. In brief, the median age was 59 (±13.6) years with a majority of female patients (47, 74.6%); 22 (35%) patients had BMI > 25 kg/m^2^; 61 (96.8%) patients had iCCA; 60 (95.2%) patients had metastatic disease at diagnosis; 47 (74.6%) patients had 2 or more metastatic sites, particularly with liver (76.2%), lymph node (55.5%), and lung (52.4%) involvement.

**TABLE 1:** Clinical characteristics of patients in the overall PEMIBIL and PEMIREAL cohort.

Demographics and disease characteristics	Overall	PEMIBIL cohort	PEMIREAL cohort
	(*N* = 63)	(*N* = 48)	(*N* = 15)
	*N* (%)	*N* (%)	*N* (%)
Age mean (years)	59	59	58
Sex			
Female	47 (74.6)	35 (72.9)	12 (80)
Male	16 (25.3)	13 (27.1)	3 (20)
ECOG performance status			
0	24 (38.1)	17 (35.4)	7 (46.7)
1	27 (42.8)	19 (39.6)	8 (53.3)
2	11 (17.4)	11 (22.9)	0
Comorbidities			
BMI ≥ 25 kg/m^2^	22 (35)	20 (41.7)	2 (13.3)
Viral hepatitis	4 (6.3)	2 (4.2)	2 (13.3)
Smoking	6 (9.5)	6 (12.5)	—
Diabetes	7 (11.1)	7 (14.6)	—
Cholangiocarcinoma location			
Intrahepatic	61 (96.8)	47 (97.9)	14 (93.3)
Extrahepatic (proximal/perihilar)	1 (1.6)	0	1 (6.6)
Missing data	1 (1.6)	1 (2.1)	0
Grading			
Well differentiated	11 (17.5)	11 (22.9)	0
Moderately differentiated	23 (36.5)	20 (41.7)	3 (20)
Poorly differentiated	10 (15.9)	7 (14.6)	3 (20)
Missing data	19 (30.1)	10 (20.8)	9 (60)
Tumour size (cm), Mean	8	8	NA
FGFR2 fusion partner			
BICC1	20 (31.7)	15 (31.2)	5 (33.3)
Disease stage at systemic treatment			
Locally advanced	3 (4.8)	3 (6.2)	0
Metastatic	60 (95.2)	45 (93.8)	15 (100)
Number of metastatic sites			
Locally advanced	3 (4.8)	3 (6.2)	0
1	13 (20.6)	9 (18.8)	4 (26.7)
2	24 (38.1)	17 (35.4)	7 (46.7)
≥ 3	23 (36.5)	19 (39.6)	4 (26.6)
Sites of disease			
Liver	48 (76.2)	37 (77.1)	11 (73.3)
Lymph nodes	35 (55.5)	27 (56.2)	8 (53.3)
Lung	33 (52.4)	25 (52.1)	8 (53.3)
Ascites	14 (22.2)	11 (22.9)	3 (25)
Other	13 (20.6)	12 (25)	1 (6.6)
Previous surgery with curative intent for early disease^a^	14 (22.2)	9 (18.7)	5 (33.3)
Previous locoregional treatment (TARE or RT or HAIC) for locally advanced or metastatic disease	6 (9.5)	6 (12.5)	0
Number of previous systemic treatment for locally advanced or metastatic disease			
1	40 (63.5)	29 (60.4)	11 (73.3)
2	13 (20.6)	10 (20.8)	3 (20)
≥ 3	10 (15.9)	9 (18.7)	1 (6.7)

^a^ 2 metastatic patients performed palliative surgery.

### 3.2 Molecular Assessment

In the whole cohort, patients were most commonly tested for *FGFR* alterations using DNA NGS platforms (31/63 patients, 49.2%; predominantly FoundationOne CDx platform in 26 patients, or others in 5 patients) or NGS fusion platforms (28/63 patients, 44.4%; Archer Fusion Plex NGS assay in 27 patients, other in 1 patient). Three (4.8%) patients were analysed using RNA sequencing, while the method used was not available for one patient.

*BICC1* was the most frequent fusion/rearrangement partner of *FGFR2* (31.7%), followed by *KIAA12* (4.8%) and others in a lower percentage, which were almost unique for each patient.

**FIGURE 1:**
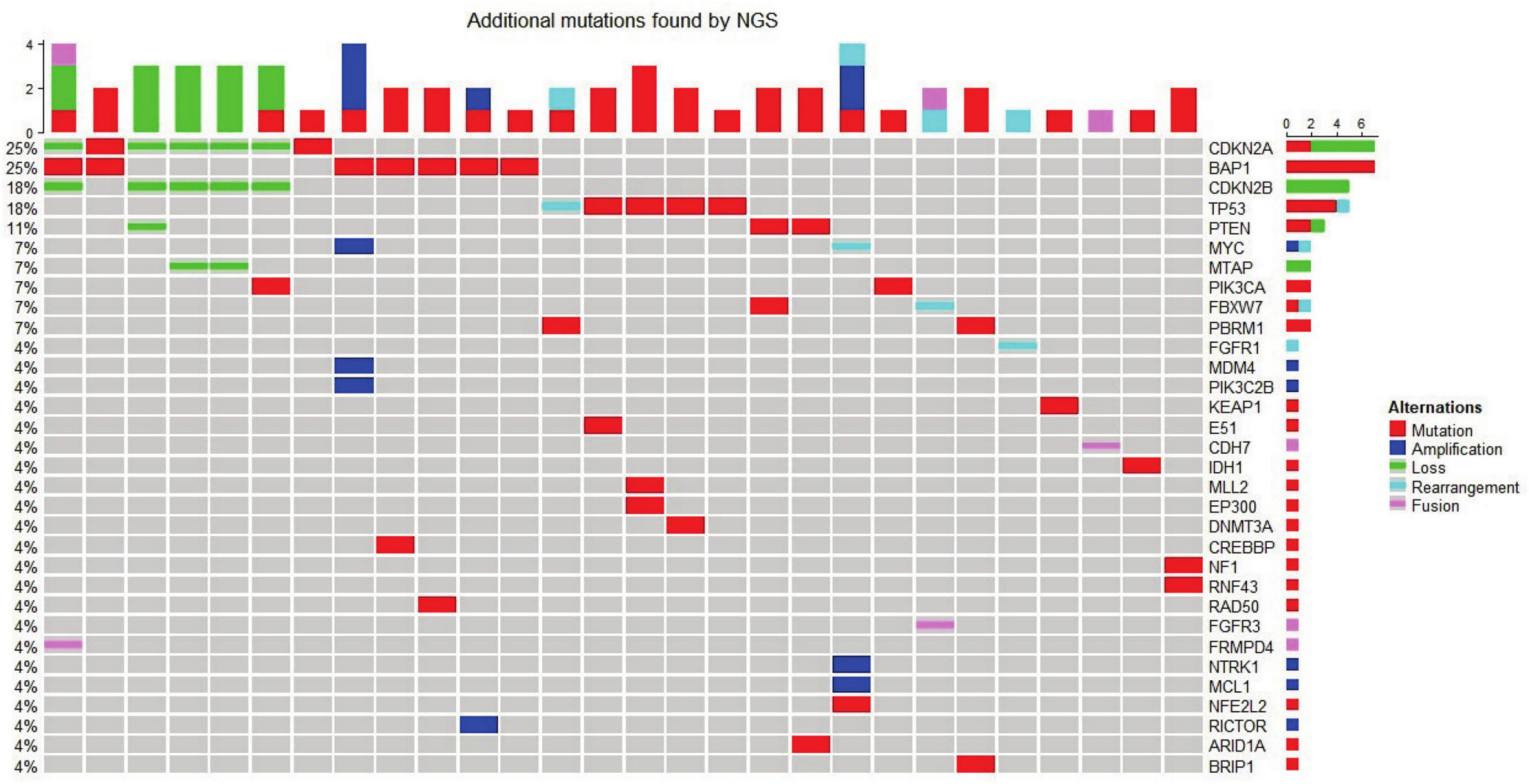
Clinicogenomic analysis of genomic co‐alterations. Oncoprint representation of GAs in patients, ordered by frequency.

The most frequent (> 5%) concomitant molecular alterations were: *BAP1* mutation (7/63, 11.1%), *TP53* mutation (6/63, 9.5%), *CDKN2A* loss or mutation (7/63, 11.1%), *CDKN2B* loss (5/63, 7.9%). Concomitant *IDH1* mutation was present in one patient. All the other available individual GAs are described in Figure 1 and in Table S1.

### 3.3 Predictive and Prognostic Role of GAs

We investigated the relationship between clinical response to pemigatinib and underlying molecular profiles, focusing on the potential role of co‐occurring alterations in primary resistance.

**FIGURE 2:**
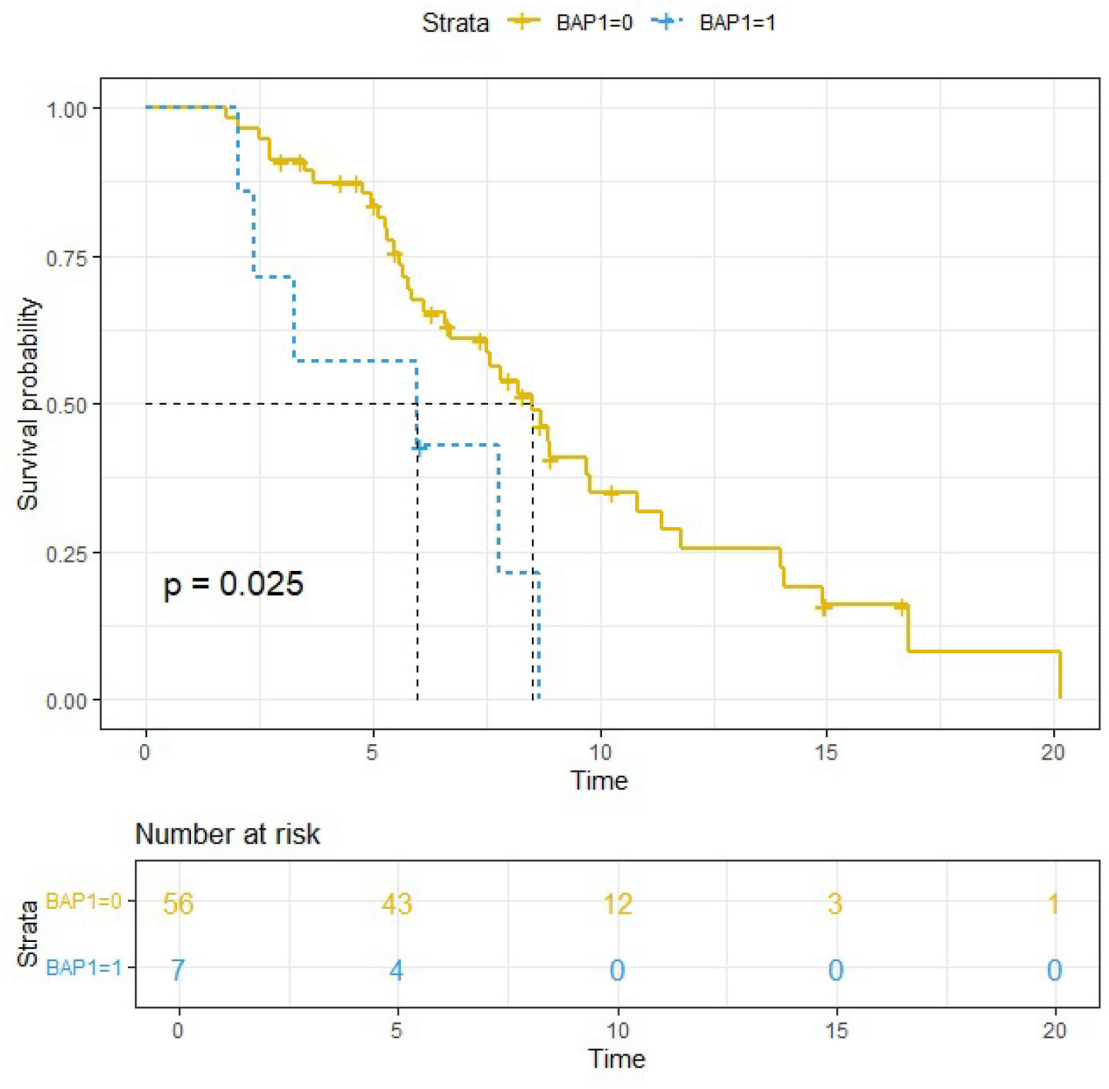
Kaplan–Meier plot showing PFS in patients with BAP1 alterations (blue line) and those without BAP1 alterations (yellow line).

No statistically significant differences were observed in ORR and DCR across the GAs analysed (Table S2). However, *BAP1*, the most frequently co‐occurring GA, had a notable impact on mPFS. Patients with *BAP1*‐mutated tumours exhibited a shorter mPFS compared to those with *BAP1* wild‐type tumours (5.97 vs. 8.52 months, *p* = 0.025, HR 2.55, 95% CI: 0.72–9.00; Figure 2). No significant differences were found in OS (*p* = 0.51, HR 1.43, 95% CI: 0.42–4.88; Figure S2). Table S3 summarizes the main patient and disease characteristics of patients with and without BAP1 mutations.

**FIGURE 3:**
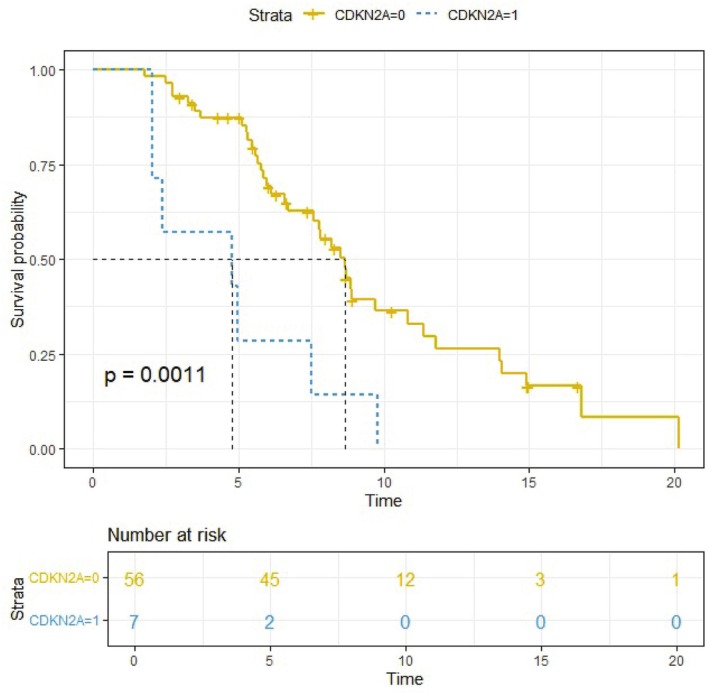
Kaplan–Meier plot showing PFS in patients with CDKN2A alterations (blue line) and those without CDKN2A alterations (yellow line).

**FIGURE 4:**
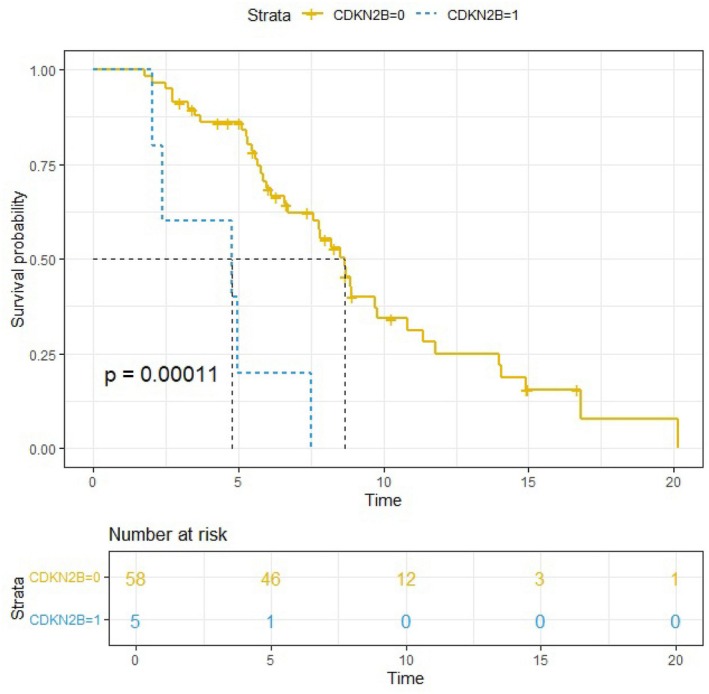
Kaplan–Meier plot showing PFS in patients with CDKN2B alterations (blue line) and those without CDKN2B alterations (yellow line).

Similarly, patients with *CDKN2A/2B* alterations had a significantly shorter mPFS. Median PFS was 4.79 months in *CDKN2A* mutant compared to 8.66 months in *CDKN2A* wild‐type patients (*p* = 0.0011, HR 3.48, 95% CI: 0.91–13.24; Figure 3), respectively. Likewise, *CDKN2B*‐mutant patients showed a shorter mPFS (4.79 vs. 8.66 months, *p* = 0.0011, HR 5.09, 95% CI: 0.75–34.36; Figure 4). In contrast, no differences in OS were observed for either *CDKN2A* (*p* = 0.71, HR 1.26, 95% CI: 0.33–4.77) or *CDKN2B* (*p* = 0.97, HR 1.03, 95% CI: 0.26–4.51), as shown in Figures S3 and S4, respectively.

**FIGURE 5:**
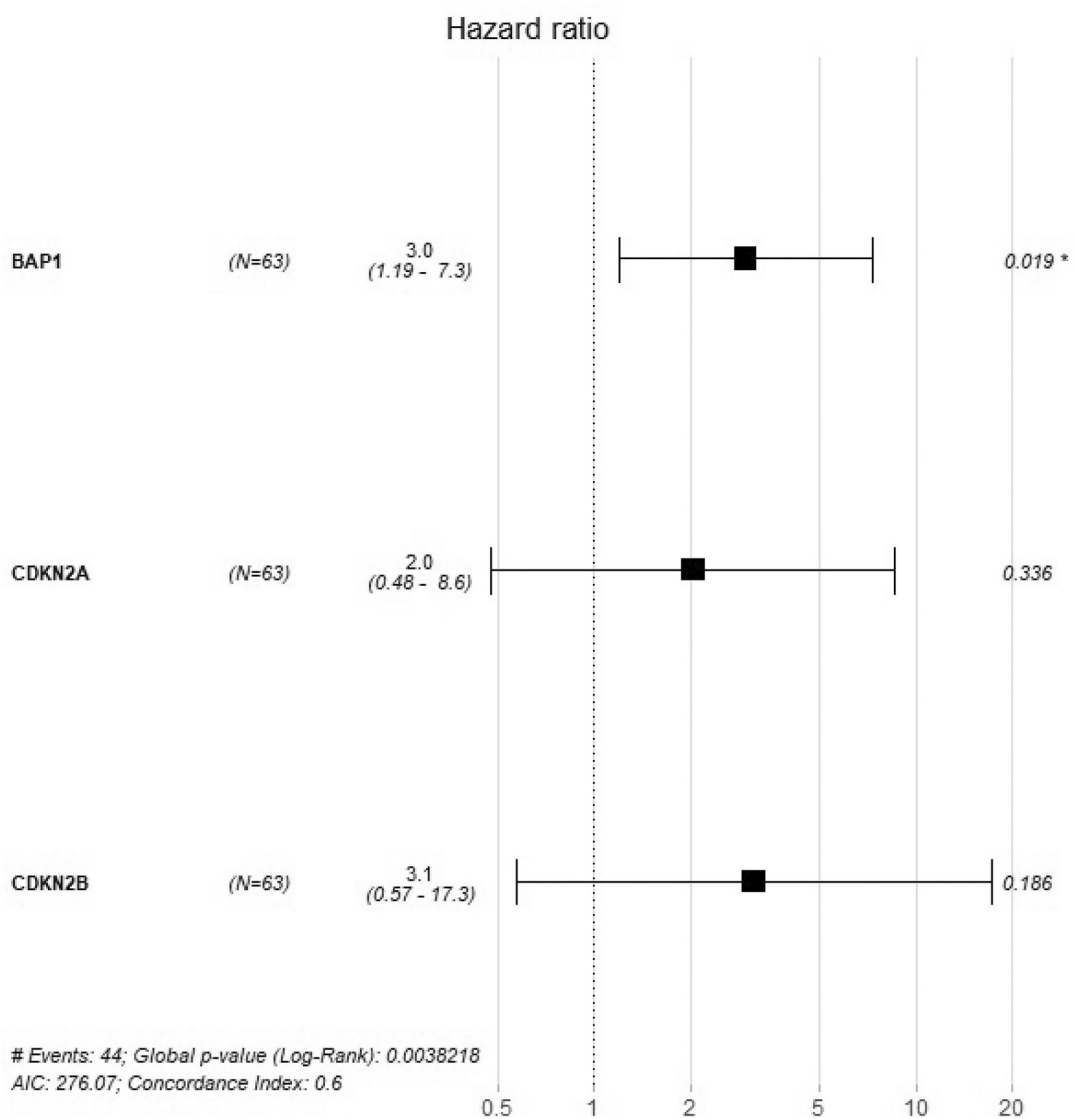
Impact of *BAP1*, *CDKN2A* and *CDKN2B* mutations on PFS: Results from multivariate analysis.

Multivariate analysis confirmed an independent statistically significant impact on PFS only for *BAP1* mutations (*p* = 0.019 HR 3.0, 95% CI: 1.19–7.3). On the other hand, *CDKN2A* and *CDKN2B* mutations lost their independent role as prognostic factors. Results of multivariate analysis can be found in Figure 5.

**FIGURE 6:**
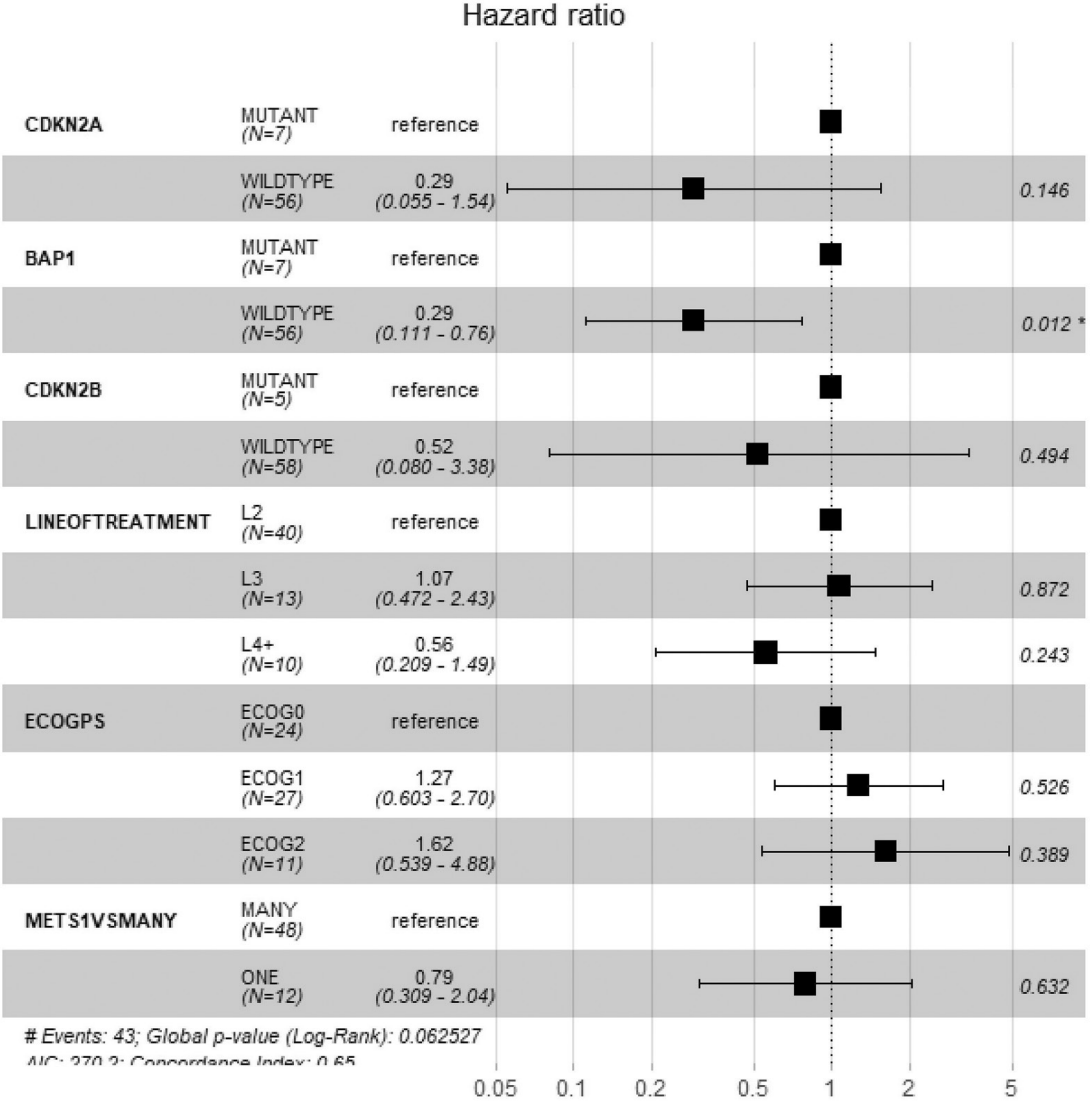
Impact of *BAP1*, *CDKN2A* and *CDKN2B* mutations together with clinical and tumour‐related prognostic factors on PFS: Results from multivariate analysis. Clinical/tumour‐related factors examined included ECOG performance status (0 vs. 1 vs. 2), treatment line (second‐line vs. third‐line vs. fourth‐line or later), and metastatic burden (single‐site vs. multi‐site metastatic disease).

Established clinical and tumour‐related prognostic factors were evaluated in multivariate models together with *BAP1*, *CDKN2A* and *CDKN2B*. The factors assessed included ECOG performance status, treatment line and metastatic burden (single‐site versus multiple‐organ involvement). For PFS, *BAP1* remained independently associated with outcome and retained statistical significance in the multivariate analysis (*p* = 0.012, HR 0.29, 95% CI: 0.11–0.76), as presented in Figure 6. In the analysis of OS, none of the molecular variables remained statistically significant after adjustment. However, ECOG performance status retained its prognostic value (Figure S5). We also evaluated patients with *TP53* (*n* = 5) and *PTEN* (*n* = 3) alterations, but no statistically significant differences in PFS or OS were detected, potentially due to the small sample size in these subgroups. Kaplan–Meier regarding *TP53* and *PTEN* can be found in Figures S6 and S7, respectively.

Overall, 20 patients harboured at least one mutation in a tumour suppressor gene, including *BAP1*, *CDKN2A/B*, *TP53*, *PBRM1*, *ARID1A*, or *PTEN*. The mPFS in patients with at least one tumour suppressor mutation was 6.74 months, compared to 8.69 months in those without mutations (*p* = 0.11, HR 1.61, 95% CI: 0.84–3.06; Figure S8A). The mOS for patients with at least one tumour suppressor gene mutation was 17.1 months, compared to not reached (NR) in patients without such mutations (*p* = 0.13, HR 1.88, 95% CI: 0.77–4.55; Figure S8B).

## 4 Discussion

The identification of *FGFR2* as a key player in cholangiocarcinoma has profoundly impacted the natural history of the disease. Although phase II trials have yielded generally promising results, a thorough assessment of clinical data—complemented by real‐world evidence—indicates that deep and durable responses to FGFR‐targeted therapies are not universally achieved. Advancing our understanding of both primary and acquired resistance mechanisms will be crucial to maximizing the therapeutic potential of FGFR inhibition.

Primary resistance appears to be influenced by co‐occurring genomic alterations, although the associations between response and mutational profiles remain speculative at this stage. In our study, while no significant differences were observed in ORR and DCR across the molecular subgroups, our findings suggest that specific alterations, particularly involving tumour‐suppressor genes such as *BAP1*, *CDKN2A* and *CDKN2B*, may be associated with worse PFS and could therefore serve as negative prognostic biomarkers in this setting.

In our cohort, *BAP1* emerged as one of the most frequent co‐mutations (11.1%), consistent with existing literature [14, 16]. Biologically, *BAP1* (*BRCA1*‐associated protein 1) is a tumour suppressor gene encoding a deubiquitinase enzyme involved in chromatin remodelling, DNA double‐strand break repair, cell cycle regulation and apoptosis [16]. Loss‐of‐function mutations in *BAP1* can lead to epigenetic dysregulation and increased genomic instability, promoting tumour progression and potentially resistance to therapy [17]. In *FGFR2*‐rearranged CCA, *BAP1* mutations may confer a more aggressive phenotype that is less responsive to FGFR inhibition, possibly through the activation of parallel oncogenic pathways or impaired apoptotic signalling. This mechanistic hypothesis is supported by findings in other malignancies, such as uveal melanoma [18], where *BAP1* loss has similarly been associated with poor prognosis and therapeutic resistance.

In our real‐world analysis, *BAP1* mutation was associated with a significantly shorter PFS and this co‐alteration retained statistical significance in multivariate analysis, after adjustment for both molecular and clinical prognostic variables. The strong association between *BAP1* alterations and PFS suggests that this molecular subgroup may represent a distinct prognostic category among patients with FGFR2‐positive cholangiocarcinoma treated with pemigatinib. In contrast, the absence of an independent association with OS after multivariable adjustment suggests that the prognostic significance of molecular alterations is more pronounced for disease control than for long‐term survival, which is likely affected by subsequent therapies and additional clinical variables. In alignment with previous studies, ECOG performance status remained an independent predictor of OS, highlighting the critical role of patient‐related factors in determining overall outcomes.

The finding of the negative prognostic role of *BAP1* contrasts with previously published data. Jain et al. [16] found no significant correlation between *BAP1* mutation status and OS in a multicentre retrospective study including 377 patients, of whom 95 harboured *FGFR2* gene fusion/rearrangement. Among these, only 42% received FGFR‐targeted therapy, limiting the ability to evaluate the role of these alterations in response or resistance to FGFR inhibition. Furthermore, outcomes such as PFS, ORR, and duration of response were not specifically analysed, making direct comparison with our results difficult.

In contrast, the study by Silverman et al. provided a more focused clinicogenomic analysis of Cohort A from the FIGHT‐202 trial. This cohort included 107 patients with centrally confirmed *FGFR2* fusions or rearrangements, treated with pemigatinib after having performed comprehensive genomic profiling via FoundationOne platform. In their analysis, *BAP1* mutations were not associated with any significant differences in OS, ORR, or median PFS.

Our findings regarding *CDKN2A* and *CDKN2B* loss also add to the growing evidence supporting their negative predictive value. Both alterations were significantly associated with reduced PFS in our cohort, in line with the results from Silverman et al. (*p* = 0.03), and consistent with the observations by Jain et al. [16], who reported significantly shorter OS (*p* = 0.4) in *FGFR2*‐rearranged patients with *CDKN2A/B* loss, although PFS was not assessed in that study.

We also assessed the clinical impact of *TP53* (*n* = 5) and *PTEN* (*n* = 3) alterations within our cohort. However, no statistically significant differences in PFS or OS were observed in these subgroups. This lack of significance is likely attributable to the limited number of cases, which constrains the statistical power to detect meaningful associations. It is well established that the frequency of co‐occurring GAs tends to be lower in *FGFR2*‐rearranged cholangiocarcinomas compared to unselected iCCA populations, further limiting the opportunity to robustly evaluate the prognostic or predictive relevance of rarer events such as *TP53* or *PTEN* mutations. In this context, the relative paucity of co‐occurring alterations suggests that tumour growth in these cases may be predominantly driven by the FGFR2 fusion itself. This could indicate a higher level of oncogenic addiction to the FGFR2 signalling pathway, potentially explaining the more pronounced impact of FGFR inhibition on PFS observed in patients lacking additional driver events. Nonetheless, emerging data suggest that some of these alterations may be clinically relevant when present. Notably, in the study by Silverman et al., *TP53* mutations (*n* = 9) were associated with markedly inferior outcomes, including a complete absence of objective responses and a significantly shorter median PFS (2.8 months vs. 9.0 months; *p* = 0.0003).

To further contextualize our findings, we looked beyond pemigatinib to other FGFR inhibitors. In the FOENIX‐CCA2 trial [19, 20]—a phase II study investigating futibatinib in previously treated patients with *FGFR2*‐altered intrahepatic cholangiocarcinoma—103 patients were included in the efficacy analysis. No significant associations were found between clinical response and specific *FGFR2* fusion partners or other concurrent genomic alterations. However, in the subgroup of 93 patients with extended 324‐gene panel sequencing, *BAP1* was the most frequent co‐alteration (43%), yet did not impact PFS (median 9.0 vs. 8.0 months; *p* = 0.7). In contrast, *CDKN2A* and *CDKN2B* deletions were detected in 22% and 17% of cases, respectively. While *CDKN2A* loss showed a trend towards shorter PFS (4.9 vs. 9.7 months; *p* = 0.2), *CDKN2B* loss was significantly associated with worse outcomes (PFS 4.8 vs. 11.0 months; *p* = 0.03). No significant differences in efficacy were observed for *TP53*‐mutated tumours.

Given the potential for functionally overlapping roles among various tumour‐suppressor genes, we extended our analysis to investigate whether alterations in any of the following well‐characterized tumour suppressors—*BAP1*, *CDKN2A/B*, *TP53*, *PBRM1*, *ARID1A* or *PTEN*—were collectively associated with clinical outcomes. Specifically, we compared PFS and OS between patients with at least one alteration in these genes and those without such mutations. In our cohort, no statistically significant differences in survival outcomes were found between these groups. This contrasts with findings from Silverman et al. [14], who reported a significantly shorter median PFS in patients with tumour‐suppressor gene loss compared to those without (6.8 vs. 11.7 months; *p* = 0.0003), suggesting that the cumulative impact of tumour‐suppressor alterations may contribute to primary resistance or more aggressive tumour biology in the context of FGFR inhibition.

Additionally, within our dataset, we identified one case of co‐occurring *IDH1* mutation (exon 4) and *FGFR2–CTNNA3* fusion, a combination typically considered mutually exclusive in iCCA [9]. The patient experienced a partial response under pemigatinib therapy. Molecular profiling was performed using an RNA‐based Fusion Plex NGS assay. This unusual co‐mutation pattern raises questions about underlying tumour heterogeneity, potential cooperative oncogenic pathways, and the dynamics of clonal evolution. Also, in Silverman et al. alterations in *IDH1* were less frequent in *FGFR2*‐rearranged patients (5.1% vs. 10.7%), but were not significantly mutually exclusive.

Taken together, our findings underscore the complexity of evaluating the prognostic and predictive roles of GAs in real‐world, moderately sized cohorts. The interpretation of genomic‐clinical correlations remains dynamic and is influenced by several key factors. First, prospective data from genetically characterized CCA patients are still limited, restricting our ability to draw definitive conclusions. While retrospective analyses such as ours contribute valuable insights, they inherently lack the robustness of prospective validation. Second, although 96.8% of patients in our cohort had iCCA, the inclusion of a small number of patients with other anatomical subtypes may have introduced some degree of biological heterogeneity. This could influence both the prognostic and predictive impact of specific genomic alterations. Furthermore, the limited number of patients harbouring certain co‐mutations reduced the statistical power of our subgroup analyses, potentially masking clinically meaningful associations. Third, the role of individual or co‐occurring genetic alterations in mediating primary resistance to FGFR‐targeted therapies remains incompletely understood. This challenge is further complicated by intratumoral heterogeneity, which may lead to underrepresentation or misinterpretation of resistance‐driving subclones. Moreover, recent evidence suggests that specific genomic events, such as BAP1 mutations, may contribute to secondary resistance mechanisms, underscoring the importance of distinguishing between primary and acquired resistance processes.

Our study also has several notable limitations. Chief among them is its retrospective design, which is susceptible to both selection and reporting biases. Furthermore, there was considerable variability in the genomic platforms used across participating centres. Both DNA‐ and RNA‐based NGS assays were utilized, differing in panel size, depth of coverage and sensitivity. DNA‐based NGS can detect a wide range of genomic alterations – including single‐nucleotide variants (SNVs), copy number variations, structural rearrangements, tumour mutational burden (TMB), and microsatellite instability (MSI)‐ but its performance depends on panel design and target regions. RNA‐based NGS is particularly effective for detecting gene fusions, alternative splicing – alterations that may go undetected by DNA‐based methods‐ and gene expression, though it is generally less sensitive to low‐frequency SNVs. Unlike DNA‐based methods, which require computational inference to identify fusion partners, RNA assays can directly identify fusion transcripts [21]. Although an integrative approach combining both DNA and RNA profiling represents the ideal standard for detecting *FGFR2* gene fusion/rearrangement and relevant co‐mutations, such comprehensive testing was not consistently available across our cohort. The lack of centralized genomic reanalysis further limits the consistency of variant interpretation and the ability to control for platform‐specific discrepancies. Standardization of NGS methodology and annotation practices would be essential for more reliable biomarker‐based stratification in future studies.

Lastly, the generalizability of our findings may be limited by the variability in NGS access across clinical settings. In some institutions, *FGFR2* gene fusion/rearrangement is still assessed using non‐NGS techniques such as FISH combined with hotspot NGS testing targeting only a limited number of genes, which may preclude comprehensive genomic profiling at the time of treatment initiation. This disparity highlights a broader issue in the real‐world application of precision oncology and underscores the need to improve equitable access to molecular diagnostics.

## 5 Conclusions

Our study provides real‐world insights into the molecular landscape of *FGFR2*‐rearranged CCA and highlights the clinical relevance of concurrent genomic alterations in shaping treatment outcomes with FGFR inhibitors. We identified *BAP1*, *CDKN2A* and *CDKN2B* co‐alterations as potential negative prognostic factors, with a significant impact on progression‐free survival. While previous studies have reported mixed findings, our data suggest that specific tumour‐suppressor gene losses may contribute to primary resistance to FGFR‐targeted therapies.

However, the rarity of certain co‐mutations, the retrospective design, and platform heterogeneity limit the strength of definitive conclusions. These findings emphasize the urgent need for prospective, standardized, and collaborative efforts to validate prognostic biomarkers and better define resistance mechanisms. Ultimately, a deeper molecular understanding of CCA will be essential to guide personalized therapeutic strategies, identify rational combination approaches, and improve outcomes for this difficult‐to‐treat patient population.

## Author Contributions

Conceptualization: C.L., R.G. and A.P. Data collection, acquisition and curation: all authors. Formal analysis: R.G., A.P., N.F. and B.D. Funding acquisition: A.P. and R.G. Investigation: all authors. Methodology: C.L., R.G., A.P. and N.F. Project administration: A.P. and N.F. Resources: A.P. and N.F. Software: R.G., A.P. and N.F. Supervision: N.F. and R.B. Validation: all authors. Writing – original draft: C.L., R.G., A.P. and R.B. Writing – review and editing: all authors. Final approval of the manuscript: all authors.

## Funding

This project was supported by a grant from Incyte Biosciences Italy S.R.L.

## Ethics Statement

This study was conducted in accordance with Good Clinical Practice guidelines, the Declaration of Helsinki, applicable local regulations, and Regulation (EU) 2016/679 on the protection of personal data. Ethical approval was obtained from the relevant committees at each participating centre. Specifically, this analysis combines data from two independent retrospective observational studies: the PEMI‐REAL study, approved by the ‘Comitato Etico Regione Marche’ in Italy in December 2022 (protocol number 325‐2022), and the PEMI‐BIL study in France (protocol number RnIPH 2022‐105), each authorized by their respective local ethics committees.

## Consent

Written informed consent to participate in the observational study was obtained from each alive patient from each cohort.

## Conflicts of Interest

Alessandro Parisi received consulting fees from AstraZeneca, Amgen, MSD, Incyte, Taiho Oncology; travel support from Merck, Daiichi‐Sankio, Accord. Matthieu Delaye has received honoraria or consulting fees from Servier and Eisai; travel expenses and congress fees from Merck and research funding from Servier and AstraZeneca. Anna Diana has received consulting fees and travel Support from Eli Lilly, Pfizer, Novartis, Roche, Gentili, Amgen, Daichii‐Sankyo, Gilead, Ipsen. Monica Niger reports travel expenses from AstraZeneca, speaker honoraria from Accademia della Medicina and Incyte; honoraria from Sandoz, Medpoint, Incyte, AstraZeneca, and Servier for editorial collaboration; and consultant honoraria from EMD Serono, Basilea Pharmaceutica, Incyte, MSD Italia, Servier, Astra Zeneca, and Taiho. David Tougeron received consulting fees from AstraZeneca, Amgen, MSD, BMS, Takeda, Beone, Gilead, Pierre Fabre, Jazz Pharmaceuticals, Servier, Incyte, Roche, and Taiho Oncology; travel support by Merck, AMGEN, Servier, Pierre Fabre, and Roche; and institutional research funding from Takeda, Gilead, MSD, Roche, Pierre Fabre, and Servier. Gael Roth received honoraria and accommodation from Servier, AstraZeneca, Bristol‐Myers Squibb, MSD, Amgen, Ipsen, Viatris, Accord Healthcare, Sanofi, Roche, Merck, Pierre Fabre, Netris Pharma, Incyte, Alpha Tau, Taiho, Jazz pharmaceutical, Astellas; research funding from Genoscience Pharma, Netris Pharma, PHRC‐K/InCA, Fondation ARC, Alpha Tau, AstraZeneca. Rita Balsano received lecture fees from AstraZeneca; travel expenses from Roche. All other authors declare that they have no known competing financial interests or personal relationships that could have appeared to influence the work reported in this paper.

## Supporting information


**Figure S1:** CONSORT diagram with patients' selection.
**Table S1:** Concomitant molecular alterations beyond FGFR2.
**Table S2:** ORR and DCR according to genomic alterations analysed.
**Figure S2:** Kaplan–Meier plot showing OS in patients with BAP1 alterations (blue line) and those without BAP1 alterations (yellow line).
**Table S3:** Clinical characteristics of patients with and without BAP1 mutations.
**Figure S3:** Kaplan–Meier plot showing OS in patients with CDKN2A alterations (blue line) and those without CDKN2A alterations (yellow line).
**Figure S4:** Kaplan–Meier plot showing OS in patients with CDKN2B alterations (blue line) and those without CDKN2B alterations (yellow line).
**Figure S5:** Impact of BAP1, CDKN2A and CDKN2B mutations together with clinical and tumour‐related prognostic factors on OS: Results from multivariate analysis. Clinical/tumour‐related factors examined included ECOG performance status (0 vs. 1 vs. 2), treatment line (second‐line vs. third‐line vs. forth‐line or later) and metastatic burden (single‐site vs. multi‐site metastatic disease).
**Figure S6:** (A) Kaplan–Meier plot showing PFS in patients with TP53 alterations (blue line) and those without TP53 alterations (yellow line). HR 0.88 (95% CI: 0.33–2.34). (B). Kaplan–Meier plot showing OS in patients with TP53 alterations (blue line) and those without TP53 alterations (yellow line). HR 2.88 (95% CI: 0.44–18.89).
**Figure S7:** (A) Kaplan–Meier plot showing PFS in patients with PTEN alterations (blue line) and those without PTEN alterations (yellow line). HR 1.09 (95% CI: 0.32–3.71). (B) Kaplan–Meier plot showing OS in patients with PTEN alterations (blue line) and those without PTNE alterations (yellow line). HR 2.33 (95% CI: 0.28–19.09).
**Figure S8:** (A) Kaplan–Meier plot showing PFS in patients with at least one tumour suppressor mutation including BAP1, CDKN2A/B, TP53, PBRM1, ARID1A, or PTEN (blue line) and those without alterations (yellow line). HR 1.61, 95% CI: 0.84–3.06. (B) Kaplan–Meier plot showing OS in patients with at least one tumour suppressor mutation including BAP1, CDKN2A/B, TP53, PBRM1, ARID1A, or PTEN (blue line) and those without alterations (yellow line). HR 1.88, 95% CI: 0.77–4.55.

## Data Availability

The data that support the findings of this study are not publicly available due to privacy and ethical restrictions but are available from the corresponding author upon reasonable request.
