# Supplementary material for: Prognostic Impact of Concomitant Genomic Alterations in FGFR2‐Positive Cholangiocarcinoma Treated With Pemigatinib
Source: Liver Int. 2026 Jul 24;46(8):e70813. doi: 10.1111/liv.70813 (PMC13396969; doi:10.1111/liv.70813)
Supplement: Supplementary file 1 — Figure S1: CONSORT diagram with patients' selection. Table S1: Concomitant molecular alterations beyond FGFR2. Table S2: ORR and DCR according to genomic alterations analysed. Figure S2: Kaplan–Meier plot showing OS in patients with BAP1 alterations (blue line) and those without BAP1 alterations (yellow line). Table S3: Clinical characteristics of patients with and without BAP1 mutations. Figure S3: Kaplan–Meier plot showing OS in patients with CDKN2A alterations (blue line) and those without CDKN2A alterations (yellow line). Figure S4: Kaplan–Meier plot showing OS in patients with CDKN2B alterations (blue line) and those without CDKN2B alterations (yellow line). Figure S5: Impact of BAP1, CDKN2A and CDKN2B mutations together with clinical and tumour‐related prognostic factors on OS: Results from multivariate analysis. Clinical/tumour‐related factors examined included ECOG performance status (0 vs. 1 vs. 2), treatment line (second‐line vs. third‐line vs. forth‐line or later) and metastatic burden (single‐site vs. multi‐site metastatic disease). Figure S6: (A) Kaplan–Meier plot showing PFS in patients with TP53 alterations (blue line) and those without TP53 alterations (yellow line). HR 0.88 (95% CI: 0.33–2.34). (B). Kaplan–Meier plot showing OS in patients with TP53 alterations (blue line) and those without TP53 alterations (yellow line). HR 2.88 (95% CI: 0.44–18.89). Figure S7: (A) Kaplan–Meier plot showing PFS in patients with PTEN alterations (blue line) and those without PTEN alterations (yellow line). HR 1.09 (95% CI: 0.32–3.71). (B) Kaplan–Meier plot showing OS in patients with PTEN alterations (blue line) and those without PTNE alterations (yellow line). HR 2.33 (95% CI: 0.28–19.09). Figure S8: (A) Kaplan–Meier plot showing PFS in patients with at least one tumour suppressor mutation including BAP1, CDKN2A/B, TP53, PBRM1, ARID1A, or PTEN (blue line) and those without alterations (yellow line). HR 1.61, 95% CI: 0.84–3.06. (B) Kaplan–Meier plot showing OS in pa [file LIV-46-0-s001.docx]

**Supplementary file - Figure 1.** *CONSORT diagram with patients’ selection*

**
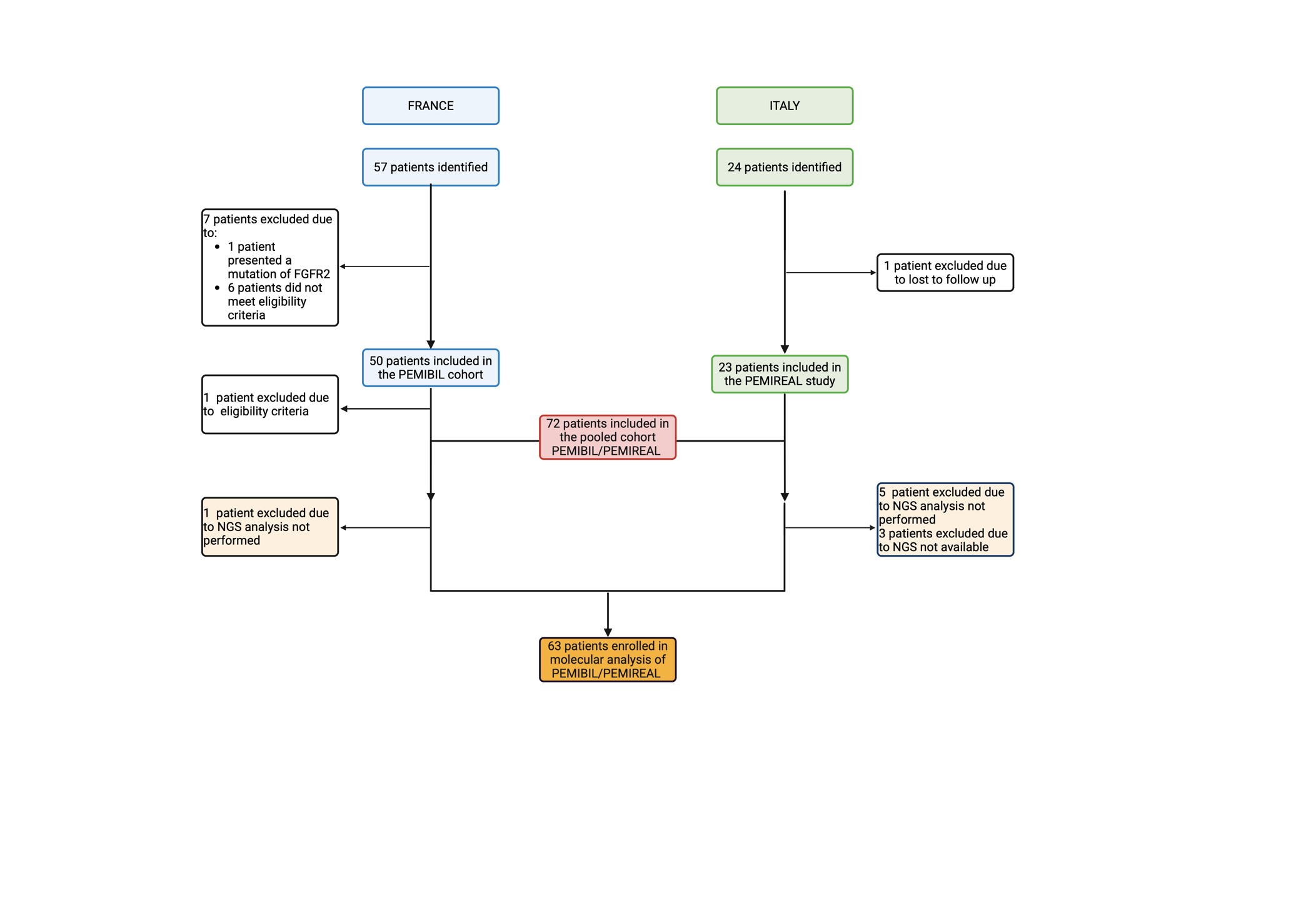
**

**Supplementary file - Table 1.** *Concomitant molecular alterations beyond FGFR2*.

| **Patients**  **28/63 (44.4%)** | **Molecular alterations** |
| --- | --- |
| Pt 1 | *FGFR1* alteration |
| Pt 2 | loss of *CDKN2A, CDKN2B, PTEN* |
| Pt 3 | amplification of *MYC, MDM4, PIK3C2B*, mutation *BAP1* |
| Pt 4 | mutation *BAP1* |
| Pt 15 | *KEAP1* mutation |
| Pt 16 | *TP53* mutation, *FGFR2* exon 11 mutation |
| Pt 23 | *CDH7-NCOA2* fusion |
| Pt 34 | *IDH1* mutation |
| Pt 39 | *CDKN2A, CDKN2B, MTAP* loss |
| Pt 40 | *PI3KCA* mutation, *CDKN2A, CDKN2B* loss |
| Pt 41 | *TP53* mutation, *MLL2* mutation, *EP300* mutation |
| Pt 42 | *DNMT3A* mutation, *TP53* mutation |
| Pt 43 | *BAP1* mutation, *CREBBP* mutation |
| Pt 44 | *CDKN2A* mutation |
| Pt 45 | *NF1, RNF43* mutation |
| Pt 46 | *BAP1, RAD50* mutation |
| Pt 47 | *PTEN, FBXW7* mutation |
| Pt 48 | *PI3KCA* mutation |
| Pt 51 | *FBW7* truncation, *FGFR3* fusion |
| Pt 52 | *TP53* mutation |
| Pt 53 | *CDKN2A* mutation, *BAP1* mutation |
| Pt 54 | *CDKN2A, CDKN2B* loss, *BAP1* mutation, *TP53* mutation |
| Pt 58 | *TP53* mutation, *PBRM1* mutation |
| Pt 59 | *NTRK1, MCL1, MYC* amplification, *MYC* rearrangement, *NFE2L2* mutation |
| Pt 63 | *RICTOR* amplification, *BAP1* mutation |
| Pt 69 | *PTEN , ARID1A , ERBB3* mutation |
| Pt 71 | *BRIP1, PBRM1* mutation |
| Pt 72 | *MTAP, CDKN2A/B* loss |

**Supplementary file – Table 2.** *ORR and DCR according to genomic alterations analyzed*

|  | Group (n) | ORR,  p value | ORR,  N (%) | DCR,  p value | DCR,  N (%) |
| --- | --- | --- | --- | --- | --- |
| **BAP1** | Altered (7) | 0.4288 | 2 (28%) | 0.02743 | 4 (57%) |
|  | Unaltered (54) |  | 27 (50%) |  | 50 (93%) |
| **CDKN2A** | Altered (7) | 0.4288 | 2 (28%) | 0.02743 | 4 (57%) |
|  | Unaltered (54) |  | 27 (50%) |  | 50 (93%) |
| **CDKN2B** | Altered (5) | 1 | 2 (40%) | 0.0963 | 3 (60%) |
|  | Unaltered (56) |  | 29 (52%) |  | 51 (91%) |
| **TP53** | Altered (5) | 0.3569 | 1 (20%) | 0.0963 | 3 (60%) |
|  | Unaltered (56) |  | 28 (50%) |  | 51 (91%) |
| **PTEN** | Altered (3) | 0.6003 | 2 (66%) | 0.3108 | 2 (66%) |
|  | Unaltered (58) |  | 31 (53%) |  | 52 (90%) |

**Supplementary file - Figure 2***. Kaplan–Meier plot showing OS in patients with BAP1 alterations (blue line) and those without BAP1 alterations (yellow line).*

**
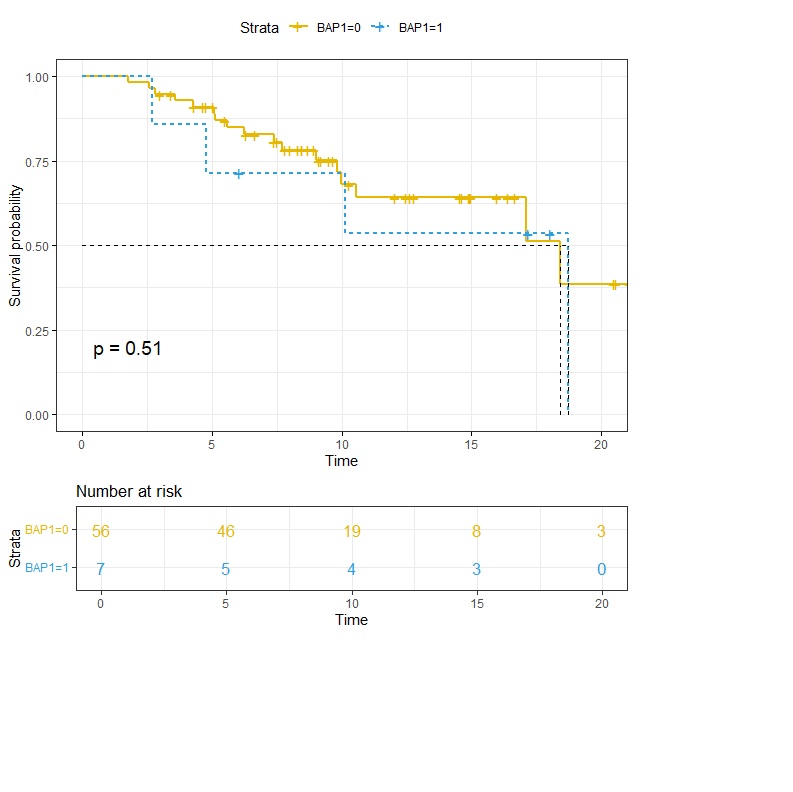
**

**Supplementary file - Table 3***.Clinical characteristics of patients with and without BAP1 mutations*.

| **Demographics and disease characteristics** | **Overall**  **(N = 63)**  **N (%)** | **BAP1-mutated**  **(N = 7)**  **N (%)** | **BAP1 wild-type**  **(N = 56)**  **N (%)** |
| --- | --- | --- | --- |
| Age mean (years) | 59 | 60.8 | 58.8 |
| Sex |  |  |  |
| Female | 47 (74.6) | 5 (71.4) | 42 (75) |
| Male | 16 (25.3) | 2 (28.6) | 14 (25) |
| ECOG performance status |  |  |  |
| 0 | 26 (41.3) | 2 (28.6) | 24 (42.9) |
| 1 | 27 (42.8) | 5 (71.4) | 22 (39.3) |
| 2 | 10 (15.9) | 0 | 10 (17.8) |
| Comorbidities |  |  |  |
| BMI ≥ 25 Kg/m^2^ | 22 (35) | 6 (85.7) | 18 (32.1) |
| Viral hepatitis | 4 (6.3) | 1 (14.2) | 3 (5.3) |
| Smoking | 6 (9.5) | 0 | 6 (10.7) |
| Diabetes | 7 (11.1) | 1 (14.2) | 6 (10.7) |
| Cholangiocarcinoma location |  |  |  |
| Intrahepatic | 61 (96.8) | 6 (85.7) | 55 (98.2) |
| Extrahepatic (proximal/perihilar) | 1 (1.6) | 1 (14.3) | 0 |
| Missing data | 1 (1.6) | 0 | 1 (0.8) |
| Grading |  |  |  |
| Well differentiated | 11 (17.5) | 0 | 11 (19.6) |
| Moderately differentiated | 23 (36.5) | 4 (57.1) | 19 (33.9) |
| Poorly differentiated | 10 (15.9) | 1 (14.3) | 9 (16.1) |
| Missing data | 19 (30.1) | 2 (28.6) | 17 (30.4) |
| FGFR2 fusion partner |  |  |  |
| BICC1 | 20 (31.7) | 3 (42.8) | 17 (30.3) |
| Disease stage at systemic treatment |  |  |  |
| Locally advanced | 3 (4.8) | 1(14.3) | 2 (3.6) |
| Metastatic | 60 (95.2) | 6 (85.7) | 54 (96.4) |
| Number of metastatic sites |  |  |  |
| Locally advanced | 3 (4.8) | 1(14.3) | 2 (3.6) |
| 1 | 13 (20.6) | 1 (14.3) | 12 (21.4) |
| 2 | 24 (38.1) | 4 (57.1) | 20 (35.7) |
| ≥3 | 23 (36.5) | 1 (14.3) | 22 (39.3) |
| Sites of disease |  |  |  |
| Liver | 48 (76.2) | 3 (42.8) | 45 (80.3) |
| Lymph nodes | 35 (55.5) | 3 (42.8) | 32 (57.1) |
| Lung | 33 (52.4) | 4 (57.1) | 29 (51.8) |
| Ascites | 14 (22.2) | 3 (42.8) | 11 (19.6) |
| Other | 13 (20.6) | 0 | 13 (23.2) |
| Previous surgery with curative intent for early disease* | 14 (22.2) | 2 (28.6) | 12 (21.4) |
| Previous locoregional treatment (TARE or RT or HAIC) for locally advanced or metastatic disease | 6 (9.5) | 0 | 6 (10.7) |
| Number of previous systemic treatment for locally advanced or metastatic disease |  |  |  |
| 1 | 40 (63.5) | 4 (57.1) | 36 (64.3) |
| 2 | 13 (20.6) | 1 (14.3) | 12 (21.4) |
| ≥3 | 10 (15.9) | 2 (28.6) | 8 (14.3) |

**Supplementary file - Figure 3***. Kaplan–Meier plot showing OS in patients with CDKN2A alterations (blue line) and those without CDKN2A alterations (yellow line).*

**
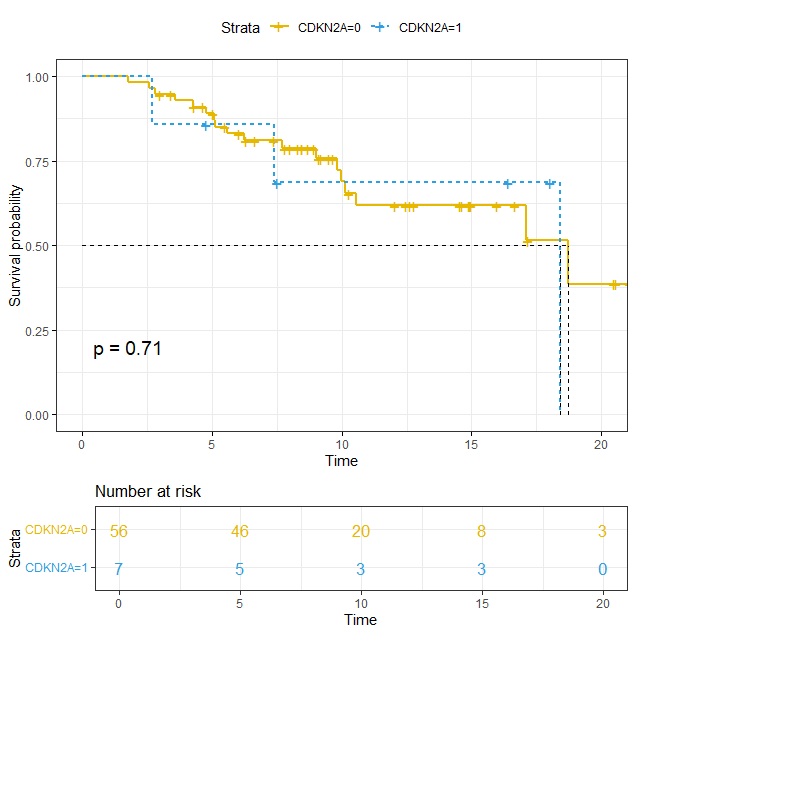
**

**Supplementary file - Figure 4***. Kaplan–Meier plot showing OS in patients with CDKN2B alterations (blue line) and those without CDKN2B alterations (yellow line).*

*
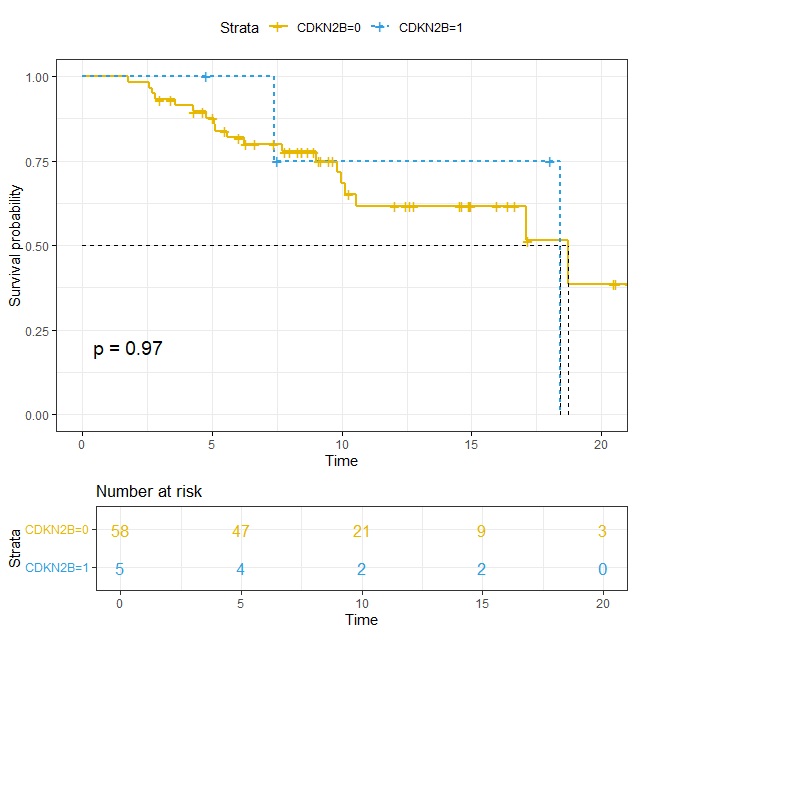
*

**Supplementary file – Figure 5. *Impact of BAP1, CDKN2A, and CDKN2B mutations together with clinical and tumor-related prognostic factors on OS: Results from multivariate analysis. Clinical/tumor-related factors examined included ECOG performance status (0 vs 1 vs 2), treatment line (second-line vs third-line vs forth-line or later), and metastatic burden (single-site vs*** *multi-site metastatic disease****).***


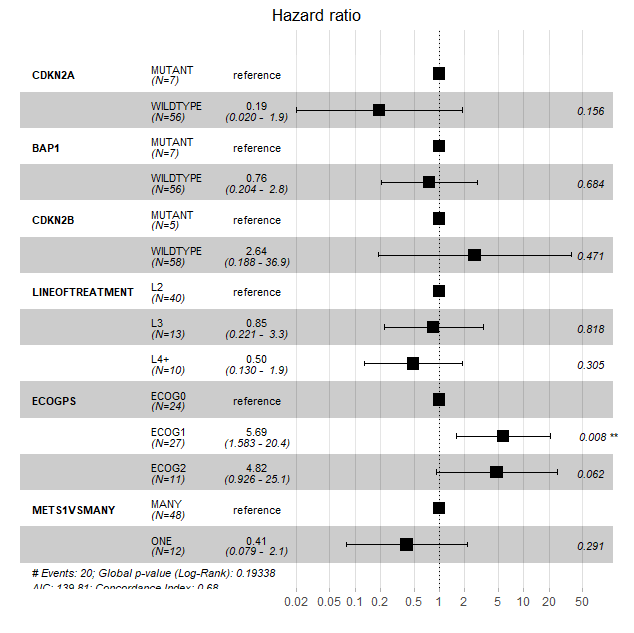


**Supplementary file - Figure 6A**. *Kaplan–Meier plot showing PFS in patients with TP53 alterations (blue line) and those without TP53 alterations (yellow line). HR 0.88 (95%CI:0.33-2.34).* **Figure 6B** *Kaplan–Meier plot showing OS in patients with TP53 alterations (blue line) and those without TP53 alterations (yellow line). HR 2.88 (95%CI:0.44-18.89)*

*
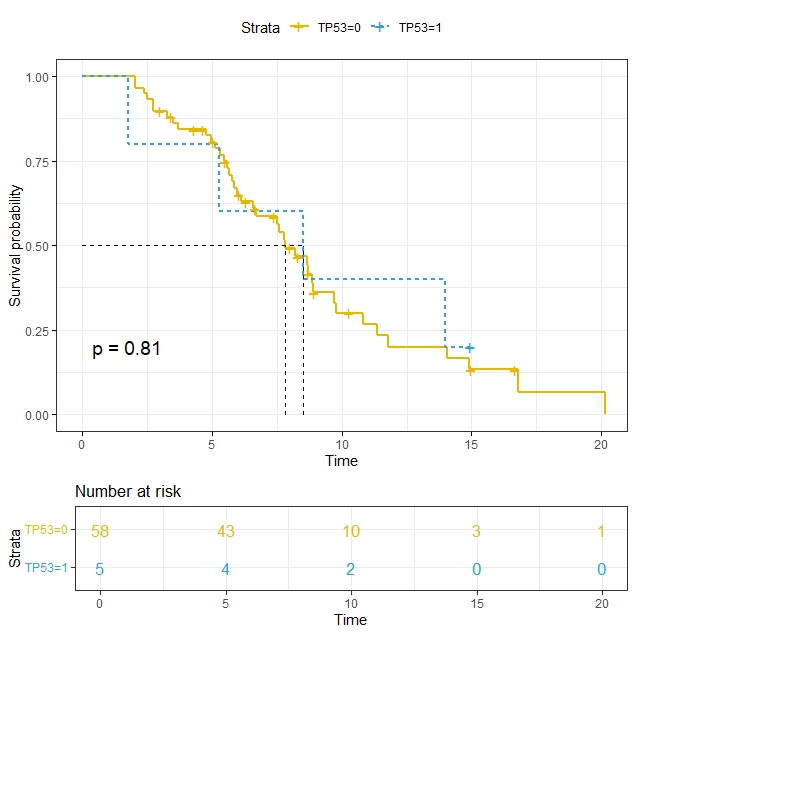
*

6A)


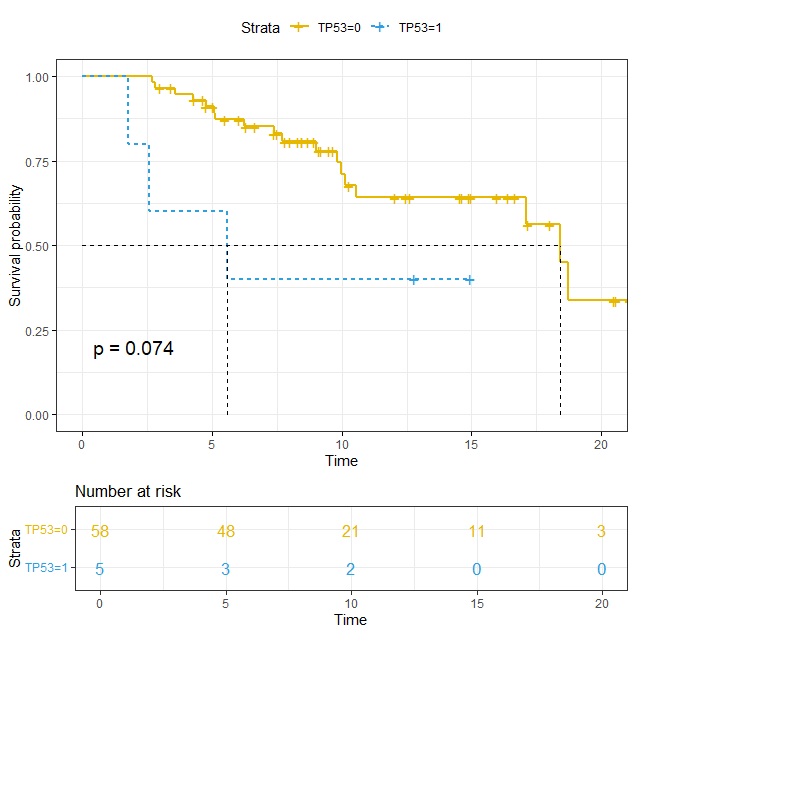


6B)

**Supplementary file - Figure 7A**. *Kaplan–Meier plot showing PFS in patients with PTEN alterations (blue line) and those without PTEN alterations (yellow line). HR 1.09 (95%CI:0.32-3.71)* ***Figure 7B*** *Kaplan–Meier plot showing OS in patients with PTEN alterations (blue line) and those without PTNE alterations (yellow line). HR 2.33 (95%CI:0.28-19.09)*


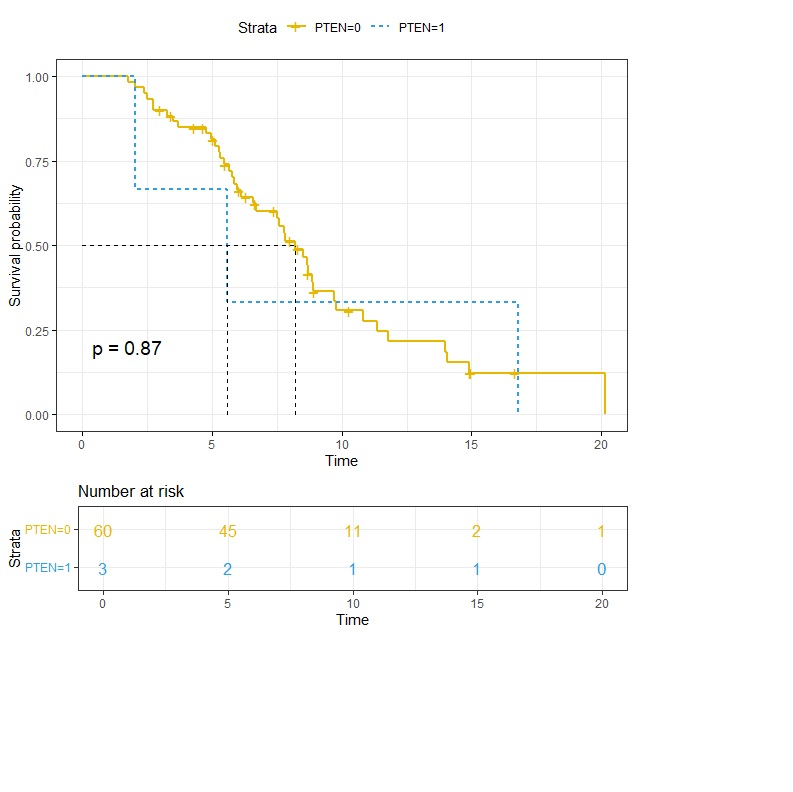


7A)


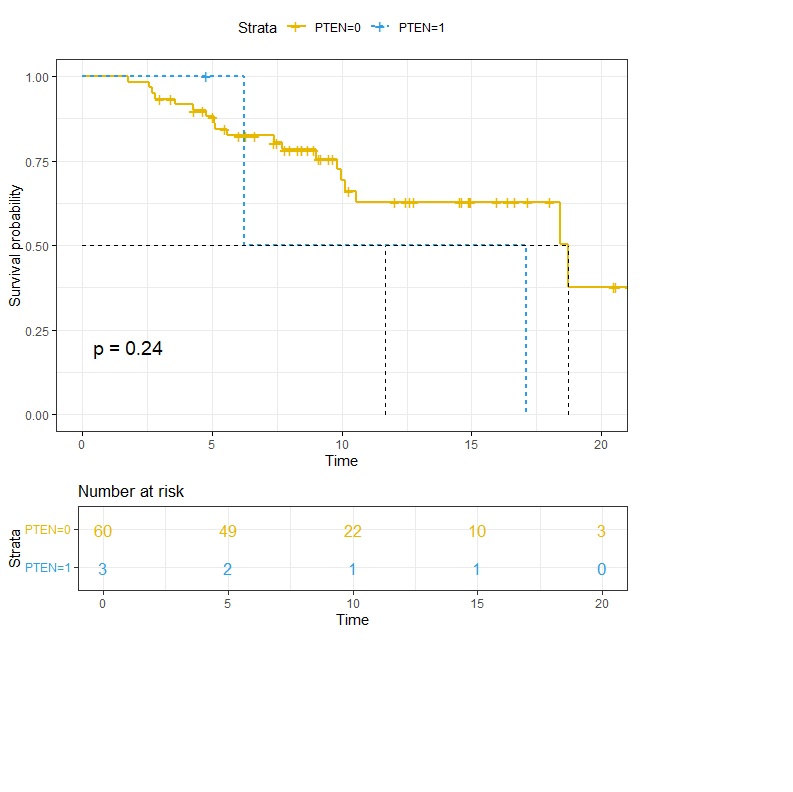


7B)

**Supplementary file - Figure 8A***. Kaplan–Meier plot showing PFS in patients with at least one tumor suppressor mutation including BAP1, CDKN2A/B, TP53, PBRM1, ARID1A, or PTEN (blue line) and those without alterations (yellow line). HR 1.61, 95%CI: 0.84–3.06.* **Figure 8B** *Kaplan–Meier plot showing OS in patients with at least one tumor suppressor mutation including BAP1, CDKN2A/B, TP53, PBRM1, ARID1A, or PTEN (blue line) and those without alterations (yellow line). HR 1.88, 95%CI: 0.77–4.55.*


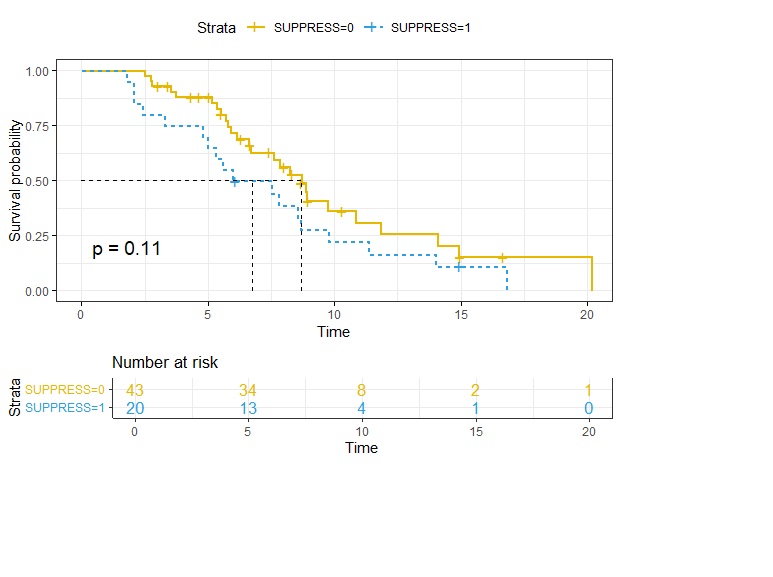


8A)


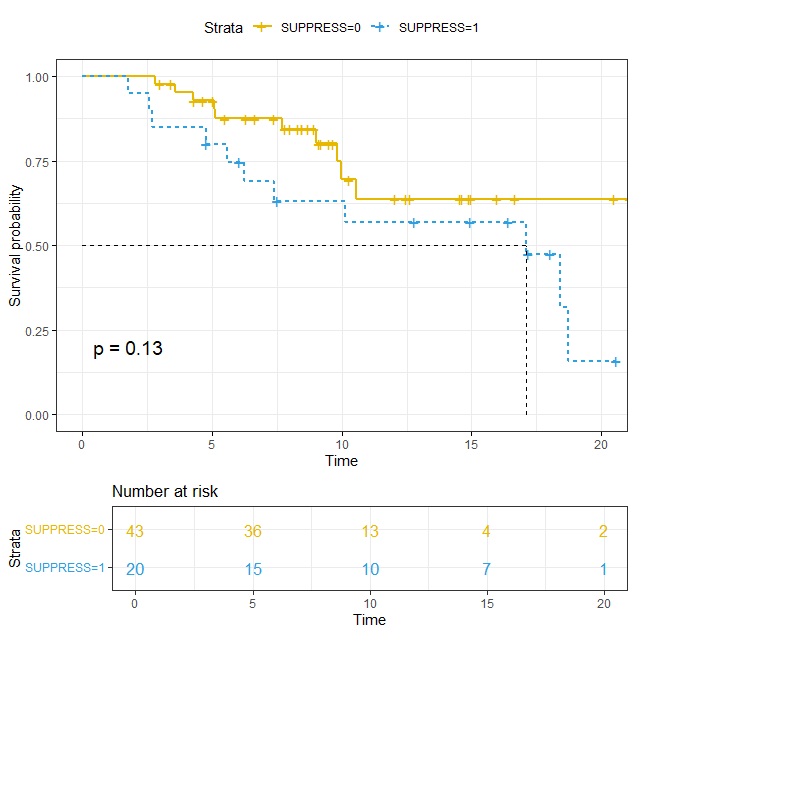


8B)
